# Aβ_42_/Aβ_40_ and Aβ_42_/Aβ_38_ Ratios Are Associated with Measures of Gait Variability and Activities of Daily Living in Mild Alzheimer’s Disease: A Pilot Study

**DOI:** 10.3233/JAD-180622

**Published:** 2018-09-25

**Authors:** Ivan Koychev, Brook Galna, Henrik Zetterberg, Jennifer Lawson, Giovanna Zamboni, Basil H. Ridha, James B. Rowe, Alan Thomas, Robert Howard, Paresh Malhotra, Craig Ritchie, Simon Lovestone, Lynn Rochester

**Affiliations:** a Department of Psychiatry, University of Oxford, UK; b Institute of Neuroscience / Institute for Ageing, Newcastle University, Newcastle, UK; c Department of Molecular Neuroscience, University College London Institute of Neurology, Queen Square, London, UK; d UK Dementia Research Institute, London, UK; e Clinical Neurochemistry Laboratory, Sahlgrenska University Hospital, Mölndal, Sweden; f Department of Psychiatry and Neurochemistry, Institute of Neuroscience and Physiology, the Sahlgrenska Academy at the University of Gothenburg, Mölndal, Sweden; g Nuffield Department of Clinical Neurosciences, University of Oxford, Oxford, UK; h Department of Biomedical, Metabolic and Neural Sciences, University of Modena and Reggio Emilia, Italy; i Center for Neurosciences and Neurotechnology, University of Modena and Reggio Emilia, Italy; j NIHR Biomedical Research Centre, University College London, UK; k Neurosciences, University of Cambridge, UK and MRC Cognition and Brain Sciences Unit, Cambridge, UK; l Department of Molecular Neuroscience, University College London; Institute of Neurology, Queen Square, London, UK; m Division of Brain Sciences, Imperial College London, UK; n University of Edinburgh, Edinburgh, UK

**Keywords:** Alzheimer’s disease, amyloid, biomarkers, cerebrospinal fluid proteins, gait

## Abstract

Gait disturbances are some of the earliest changes in dementia and their monitoring presents an opportunity for early diagnosis. The exact relationship between gait and well-established biomarkers of Alzheimer’s disease (AD) remains to be clarified. In this study we compared gait-related measures with cerebrospinal fluid (CSF) markers of AD pathology. We recruited seventeen participants with mild AD in a multi-site study and performed gait assessment as well as lumbar punctures to obtain CSF. CSF Aβ_42_/Aβ_40_ and Aβ_42_/Aβ_38_ correlated positively with measures of variability (step time and step length) in the clinic-based assessments. This was driven by a negative relationship between gait variability and Aβ_40_ and Aβ_38_ but not Aβ_42_.The amyloid ratios and gait variability measures were also associated with more severe functional impairment. We interpret these data as an indication that increasing amyloid production (i.e., increasing Aβ_40_ and Aβ_38_) is associated with diminishing cognitive-motor control of gait. These preliminary results suggest that the two amyloid ratios may be a marker of the earliest disturbances in the interplay between cognitive and motor control which characterize dementia.

## INTRODUCTION

The re-conceptualization of Alzheimer’s disease (AD) as a continuum of disorders spanning from intact cognition in the context of abnormal biomarkers of AD pathology to dementia has led to calls for a shift toward earlier diagnosis of dementia, before significant cognitive and functional impairment manifest [1]. Achieving this would allow researchers to develop a better understanding of the preclinical phase of disease. In this respect, features identifiable in clinical and home settings that occur prior to marked cognitive impairment are of particular interest and gait disturbance is potentially valuable. Walking is a complex cognitive and motor process that is sensitive to the loss of higher order cognitive control, with studies showing that gait impairment is associated with poor attention and memory [2–4]. The relevance to early diagnosis is demonstrated by studies showing gait disturbances in dementia start to develop up to 12 years before first symptoms [5]. In addition, poorer motor function and slower walking speed act as predictors for cognitively healthy individuals converting into mild cognitive impairment (MCI) [5] as well as MCI progressing to dementia [6–8], leading to the proposition that change to the variability of gait parameters is a hallmark of the earliest stages of the disease [9, 10]. The link of the observed gait disturbance to the underlying AD pathology, however, remains largely unclarified.

Accumulation of amyloid-β (Aβ) plaques is one of the pathophysiological hallmarks of AD. Quantification of Aβ in cerebrospinal fluid (CSF) is the best validated fluid biomarker for obtaining *in vivo* information on amyloid accumulation [11] with decreased levels of Aβ_42_ consistently found in AD [12]. Several shorter forms of Aβ are also present (Aβ_40_ and Aβ_38_) and evidence points to Aβ_42_/Aβ_40_ and Aβ_42_/Aβ_38_ ratios being more specific to Aβ plaque pathology than Aβ_42_ alone [13]. Low levels of Aβ_42_ in the CSF predict gait decline in other neurodegenerative disorders such as Parkinson’s disease although it is unclear whether such a link also exists in dementia [14].

In this study, we sought to clarify the relationship between gait abnormalities in AD and CSF markers of AD pathology in a group of people with clinical diagnosis of AD. We hypothesized that more severe pathology (lower CSF Aβ) will be associated with indices of worsening gait and impaired activities of daily living.

## METHODS

### Participants

Patients with mild AD were recruited from six centers of excellence in the UK as part of the pilot Deep and Frequent Phenotyping (DFP) study [15, 16]. The main inclusion criteria for the DFP study were diagnosis of AD according to NIAA criteria [17]; MMSE score above 20; age between 55 and 80; Rosen modified Hachinski ischemic score≤4; ability to walk independently for at least 15 m (see previous publication [15] for a description of the study design). Participants were assessed over a 6-month period during which they completed a battery of tests, including gait (clinic-based assessments at Day 169) assessments and cerebrospinal fluid (baseline and Day 182) analyses reported here. Dementia severity was assessed using Clinical Dementia Rating (CDR) [18] and the presence of depressive symptoms using the Geriatric Depression Scale (GDS) [19]. Level of function was evaluated using the Bristol Activities of Daily Living Scale (BADLS) [20].

### Gait procedures

Clinic-based assessments employed single- and dual-task testing conditions. For both, participants were required to walk in a straight line for 6 m (repeated 6 times) or 10 m (repeated 4 times), depending upon availability of clinic space. Under the single-task condition participants were requested to walk in a straight line only. When performing the dual task, participants walked while reciting strings of numbers, the length of which was determined by their seated digit span (forward) performance [21]. Gait outcomes were derived from a theoretical model based on a principal component analysis [22] and included a range of characteristics sensitive to cognition [23]. For the clinic-based assessments, gait characteristics were extracted based on temporal algorithms identifying initial and final contact events within the gait cycle [21].

### CSF analysis

CSF was collected at the baseline visit and 6-month follow-up. Aβ_42_, Aβ_40_, and Aβ_38_ concentrations were measured using the MSD Aβ Triplex assay (Meso Scale Discovery, Rockville, MD). Well-established standard operating protocols for sample collection and management were used [12] and assays performed according to manufacturer’s instructions, as previously described in detail [24]. All measurements were performed by board-certified laboratory technicians who were blinded to clinical data. The measurements were performed on one occasion using one batch of reagents. Intra-assay coefficients of variation were below 10% and all samples measured in the quantitative range of the assays.

### Statistical analysis

SPSS (Version 25) was used to analyze the data. The relationship between amyloid CSF measures (Aβ_42_/Aβ_40_, Aβ_42_/Aβ_38_ ratios as well as Aβ_42_, Aβ_40_, and Aβ_38_) and gait measures were compared using two-way Spearman’s correlations. For the clinic-based assessment. we examined step velocity, step length and its variability, and step time and its variability for the single- and dual-task conditions [21]. Significant correlations between gait and CSF measures were followed with Spearman’s correlations with the BALDS score, a measure of function in dementia. A threshold of *p* < 0.05 was used to guide statistical interpretation.

### Ethics

The study was approved by a National Research Ethics Committee London on 19 September 2014 (reference 14/LO/1467). All participants had mental capacity for informed consent.

## RESULTS

### Demographics and clinical measures

17 participants underwent lumbar puncture on at least one occasion (14 at baseline and 6 months, 1 completed the baseline only and 2 the follow-up only). To obtain 17 full CSF datasets, we averaged the biomarker values for those with two measurements and used the single measurement for the other participants. Mean and standard deviation values for CSF measures were as follows: Aβ_38_ 2234.2±731.7 pg/ml; Aβ_40_ 5576.8±1405.4 pg/ml; Aβ_42_ 288.4±76.6 pg/ml; Aβ_42_/Aβ_40_ 0.05±0.01 and Aβ_42_/Aβ_38_ 0.14±0.04. Of the 17 participants who had CSF sampling, 16 had the clinic-based and free-living gait testing, respectively. Mean age of the sample was 67 years (SD 7.5) and 8 of 17 were female. AD severity was mild (mean MMSE was 25, SD 2.4; mean CDR was 0.7, SD 0.2) with minimal functional impairment affecting independence (mean BADLS of 4, SD 3.4) and were not depressed (mean GDS score of 2.2, SD 1.4). See Table 1 for a summary of the demographic and clinical measures.

**Table 1:** Summary of demographic and clinical variables with mean values and standard deviations (in brackets); amyloid isomer concentrations expressed in pg/ml

	Mean (SD)
Age	67 (7.5)
MMSE	25 (2.4)
CDR	0.7 (0.2)
BADLS	4 (3.4)
GDS	2.2 (1.4)
Aβ_38_	2234.2 (731.7)
Aβ_40_	5576.8 (1405.4)
Aβ_42_	288.4 (76.6)
Aβ_42_/Aβ_40_	0.05 (0.01)
Aβ_42_/Aβ_38_	0.14 (0.04)

### Gait and Aβ relationships

**Fig. 1.**
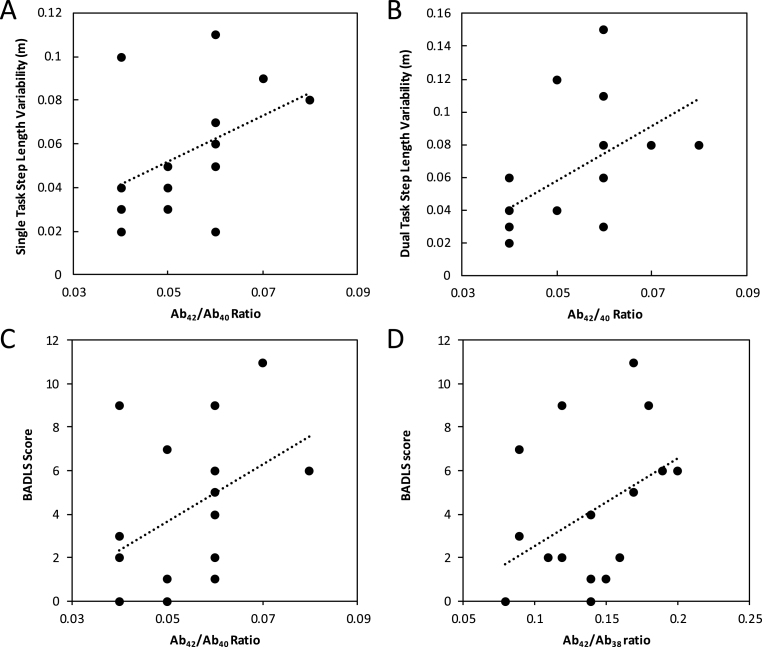
Relationships between CSF with gait (A, B) and daily function (C, D).

For both single- and dual-task conditions of the clinic-based assessment there were no significant correlations between the amyloid ratios or individual Aβ peptides and step velocity, length, and time. For the single condition task, however, the variability in step length and the variability in step time were correlated positively with Aβ_42_/Aβ_40_ and Aβ_42_/Aβ_38_ ratios. The variability in step length and the variability in step time were negatively correlated with Aβ_38_ and Aβ_40_ but not Aβ_42_ levels. Similarly, in the dual-task condition, the variability in step length and the variability in step time were correlated positively with Aβ_42_/Aβ_40_ and Aβ_42_/Aβ_38_. The variability in step length was negatively correlated with Aβ_40_ and Aβ_38_ but not Aβ_42_ levels. However, in the dual-task condition, there was no significant relationship between the variability in step time and any of the individual amyloid peptide measures (Table 2, Fig. 1A-B).

**Table 2:** Summary of study comparisons (Spearman’s Rho coefficient and *p* value in brackets, significant (*p* < 0.05) results in bold)

Gait Task	Aβ_42_/Aβ_40_	Aβ_42_/Aβ_38_	Aβ_42_	Aβ_40_	Aβ_38_
Single Task Condition
Step Velocity	–0.07 (0.80)	–0.14 (0.62)	0.25 (0.36)	0.11 (0.69)	0.23 (0.40)
Step Length	–0.01 (0.97)	–0.09 (0.74)	0.19 (0.49)	0.04 (0.88)	0.20 (0.45)
Step Length Variability	**0.60 (0.02)**	**0.60 (0.01)**	0.06 (0.82)	**–0.54 (0.03)**	**–0.55 (0.03)**
Step Time	0.14 (0.60)	0.16 (0.56)	–0.23 (0.38)	–0.18 (0.5)	–0.20 (0.45)
Step Time Variability	**0.59 (0.02)**	**0.62 (0.01)**	–0.04 (0.88)	**–0.56 (0.02)**	**–0.64 (<0.01)**
Dual Task Condition
Step Velocity	–0.02 (0.95)	–0.04 (0.88)	0.19 (0.49)	–0.02 (0.93)	0.09 (0.75)
Step Length	0.06 (0.84)	–0.02 (0.95)	0.07 (0.80)	–0.12 (0.66)	0.07 (0.81)
Step Length Variability	**0.67 (<0.01)**	**0.66 (<0.01)**	0.14 (0.59)	**–0.51 (0.04)**	**–0.54 (0.03)**
Step Time	–0.08 (0.77)	–0.12 (0.65)	–0.15 (0.57)	0.14 (0.61)	0.15 (0.58)
Step Time Variability	**0.62 (0.01)**	**0.56 (0.03)**	0.14 (0.59)	–0.4 (0.13)	–0.4 (0.13)

To assess the functional significance of the observed relationships, we correlated BADLS score with the clinic-based variability measures. BADLS correlated positively with the variability in step length (Spearman’s Rho = 0.7, *p* < 0.01 and 0.56, *p* = 0.02 for single and dual task conditions respectively) and the variability in step time (Spearman’s Rho = 0.6, *p* < 0.01 and 0.48, *p* = 0.05 for single and dual task conditions respectively). In addition, BADLS correlated positively with Aβ_42_/Aβ_40_ ratio (Spearman’s Rho = 0.53, *p* = 0.03) and Aβ_42_/Aβ_38_ ratio (Spearman’s Rho = 0.47, *p* < 0.05), but not with any of the individual amyloid peptide measures (Fig. 1C-D).

## DISCUSSION

In this pilot study, we found an association between measures of gait variability and CSF amyloid markers of AD pathology (Aβ_42_/Aβ_40_ and Aβ_42_/Aβ_38_ ratios) in a group of mild AD patients. The association was driven by negative correlations between the measures of gait variability and the individual levels of Aβ_40_ and Aβ_38_ isoforms as there was no significant association between measures of gait variability and Aβ_42_ level. We also found that both the measures of gait variability and amyloid ratios correlated with severity of impairment in activities of daily living.

The finding of an association between measures of Aβ peptides and gait variability rather than the average gait characteristics (step length, time, and velocity) is potentially interesting. Changes to measures of gait variability are common features of age-related cognitive impairment and neurodegenerative disorders. They are associated with poor mobility as well as increased risk of falls, and are a harbinger of future cognitive decline [25]. Measures of gait variability may also be associated with dementia severity and may even help distinguish between dementia subtypes [10]. However, the underlying mechanisms of increased gait variability are poorly understood. One potential theory is that the increased variability is an early compensatory mechanism, where the variability in clinical outcomes increases to account for decreasing physiological reserve. The proposed theory predicts that in later stages these compensatory mechanisms fail leading to declining variability [26]. The negative relationships between CSF Aβ_40_ and Aβ _38_ isoform levels and measures of gait variability in our sample of individuals with mild AD are consistent with this theory. However, it is unclear whether measures of gait variability will decline with increasing disease severity, as predicted by MacDonald et al. [26], or increase further with declining cognitive control of gait [9]. That the relationship between gait and Aβ_40_ and Aβ _38_ peptides was significant only in the simpler condition (single-task) relative to the more complex one (dual-task) may be due to a larger variation in response to dual-task paradigms as previously observed in older adults with and without Parkinson’s disease [27]. The findings from this small study add to the growing postmortem and *in vivo* evidence supporting a relationship between measures of gait abnormalities and changes in Aβ amyloid metabolism. In a postmortem study, the rate of decline in gait speed was associated with levels of AD pathology especially in the putamen [28]. Using PET amyloid imaging Del Campo and co-authors demonstrated an association between regional amyloid burden (putamen, occipital cortex, precuneus, and anterior cingulate) and gait speed in non-demented individuals [29]. Another study found that lower levels of Aβ_42_ were associated with gait decline in patients with Parkinson’s disease [14].

In this study we found a statistically significant negative correlation between measures of gait variability (step time and length variability) and CSF Aβ_42_/Aβ_40_ and Aβ_42_/Aβ_38_ ratios, driven by changes in Aβ_40_ and Aβ_38_ levels but not Aβ_42_. The exact significance of this is unclear, but CSF Aβ_40_ and Aβ_38_ isoform levels may represent measures of amyloid production and/or turnover rather than a reflection of sequestration into plaques as is thought to be the case for Aβ_42_. In our study, gait variability decreased with increase in Aβ_40_ and Aβ_38_ thus suggesting a relationship between true amyloid production and deterioration of the neural control of gait. In addition to differentiating between AD and other dementias [13], it appears they carry additional information about pathological burden in other conditions such Parkinson’s disease where a relationship between CSF alpha-synuclein and Aβ_40_ and Aβ_38_ rather than Aβ_42_ has been reported [30]. In another study in Parkinson’s disease, ventricular enlargement as a marker of neurodegeneration was associated with CSF Aβ_38_, Aβ_40_, and Aβ_42_ levels [31]. The lack of relationship between Aβ_42_ and gait measures is likely explained by the Aβ_42_ levels already being at a very low level (mean 288 pg/ml), thus exercising a floor effect.

The study is limited by its small sample size. A larger version of this pilot study is underway to validate these findings— the study will increase the sample size and provide longitudinal gait and CSF measurements. Another limitation is that to increase the sample size, we averaged CSF measures for 14 participants (samples taken 6 months apart) while for 3 participants we used their single measure taken either at baseline or 6 months. We made this decision on the basis of a previous analysis that found no significant change in CSF measures over this period in the same population [15].

In conclusion, this is the first study to-date to explore the link between gait and CSF amyloid measures in early AD patients. We demonstrated a relationship between measures of gait variability and Aβ_42_/Aβ_40_ and Aβ_42_/Aβ_38_ levels, which was mirrored in changes in activities of daily living function. Thus, measures of gait variability may act as an early marker of AD dementia. Further well-powered longitudinal studies are required to confirm this relationship and explore its change as the disease progresses.
